# Medical students’ knowledge and attitude towards tele-education and associated factors at University of Gondar, Ethiopia, 2022: mixed method

**DOI:** 10.1186/s12909-023-04579-5

**Published:** 2023-08-22

**Authors:** Rorisa Tadele Hailemariam, Araya Mesfin Nigatu, Mequannent Sharew Melaku

**Affiliations:** 1 Department of Health Informatics, School of Public Health, Arbaminch University, Arbaminch, Ethiopia; 2 Department of Health Informatics, Institute of Public Health, University of Gondar, Gondar, Ethiopia

**Keywords:** Knowledge, Attitude, Tele-education, Medical students, Gondar

## Abstract

**Background:**

Tele-education is the use of ICTs to conduct remote learning. It has been utilized to deliver ongoing training for many years. The world’s modern culture is increasingly reliant on the use of information technology to enhance standards of education. However, in order to deploy successful e-learning systems in a developing nation, understanding of user characteristics is required in the creation and usage of e-learning systems. Thus, this study will enable us to understand the user’s level of knowledge and attitude towards tele-education.

**Methods:**

An institution-based quantitative cross-sectional study supported by qualitative design was used 397 medical students at University of Gondar from May to June 2022. A pre-tested self-administered structured questionnaires and in-depth interview were used to collect quantitative and qualitative data respectively. Thematic-content analysis was conducted using open-code software for analyzing qualitative data. Quantitative data was entered to Epi-data version 4.6 and exported to SPSS version 25 software for further statistical analysis. Binary logistic regression was conducted. The adjusted odds ratio(AOR) was used to measure the association between the dependent and independent variables.

**Results:**

A total of 397 medical students were participated in this study with a response rate of 93.63%. In this study nearly six out of ten 230(57.9%) of study participants had good knowledge towards tele-education. More than half. 211(53.1%) of medical students participated on the study also had a favorable attitude towards tele-education. Factors associated with knowledge about tele-education is training related to ICT (AOR = 2.27 95% CI; (1.13,4.55)), knowledge of medical education digitization (AOR = 3.80 95% CI; (2.12,6.84)), high computer literacy (AOR = 2.82 95% CI; (1.68,4.72)) and favorable attitude towards tele-education (AOR = 3.52 95% CI; (2.12,5.84)). Factors associated with attitude towards tele-education is age group > 21 (AOR = 3.89, 95% CI; (1.33,11.39)) and good knowledge towards tele-education (AOR = 3.42,95%CI;(2.06,5.66)).

**Conclusion:**

The study revealed that the knowledge of the medical students was good and nearly five out of ten of them had a favorable attitude towards tele-education. The study shows that training related to ICT, knowledge of medical education digitization, high computer literacy and favorable attitude towards tele-education were associated significantly with knowledge of tele-education. In this study age group > 21 and good knowledge towards tele-education of study participants were associated significantly with attitude towards tele-education.

## Background

Tele-education is known as the use of ICTs in the delivery of remote learning, and the way in which it is provided is depending on the technology and media utilized [1]. It has been utilized to deliver ongoing training for many years [1, 2]. Any educational or learning process or system in which the teacher and instructor are separated geographically or in time from his or her learners; or in which students are isolated from other students or educational materials is known as distance learning [3].

Audio andvideo technologies are the major modality of learning and teaching. Audio technologies involve the synchronous or asynchronous transmission of the spoken word (voice) between learners and instructors. Videoconferencing, or interactive television, is called synchronous and slow-scan video, interactive videodiscs, and videotapes are examples of asynchronous instructional video technologies [4–6].

Universities need to adjust their teaching curricula for science, research, and education to keep up with the advancement of computer networks and the introduction and use of new technologies in all facets of human activity [3]. New prospects for tele-education have emerged as a result of the expansion of the Internet and the World Wide Web [2].

In Ethiopia Telemedicine systems continue to be underutilized and have not achieved their intended goals despite having adequate hardware and software facilities to conduct real-time video conference-based medical consultations, as well as to store and forward patient medical records and medical education exchange capabilities between two or more sites [7].

Despite recent improvements, the higher education industry in Ethiopia continues to face numerous obstacles to its growth and development. One of these difficulties is decreased educational quality [8].

Medical students can use tele-education to continue their education and maintain their qualifications and certifications [9]. Today’s technical advancements are so quick that only a highly skilled workforce with research and training experience in the relevant domains can analyze and adjust such technologies for local usage [10]. Over the last decade, the growth and greater usage of e-learning has revealed the effectiveness and usefulness of information technology in education [9].

Tele-education has grown in popularity in recent years; however, its use in medical schools has been inconsistent, and it appears to be more common in basic medical science courses than clinical instruction [11, 12]. A study of medical students from more than 13 Libyan medical institutions, only 21.1% agreed that e-learning could be used for clinical aspects, as compared with 54.8% who disagreed with this statement and 24% who were neutral [12]. Thus, tele-education and basic computer skills were indicated as having an influence on future medical practice as part of a career learning process [3].

In today’s world, tele-education aided teaching in surgery is a revolutionary surgical skills training system that combines distant, interactive, rising, and low-cost simulation-based education with one-on-one guidance and longitudinal evaluations with the goal of improving patient treatment quality by providing patient-centered and collaborative surgical education and coaching [13]. As a result of these improvements in the educational system, the use of information technology to enhance educational quality is now irreversible, and it is becoming an integral part of modern culture throughout the world [14–16]. But, inadequate infrastructure (gadgets and internet) and lack of experienced people to apply this platform are among the challenges of e-learning in medical education in these decade [17].

The importance of distance learning at institutions throughout the world is undeniable, but more research is required to assess its effectiveness [18]. ICT-enhanced education and increased awareness among stakeholders would have a positive impact on society. So, applying ICT in many aspects of education systems at the university level education can assist to improve the quality of education and standards as well [14].

The introduction and implementation of tele-education in medicine is expected to produce the following outcomes: advancement and incorporation of information systems technologies in medical education, establishment of flexible infrastructure that will enable all students and teaching staff to access e-Learning, increase in quality of academic population’s digital literacy, assure high educational standards for students and teaching staff, and assisting medical team in developing “Lifelong learning” [3].

There are two types of tele-education methods: synchronous, which is real-time, and asynchronous, which is recorded but not provided in real-time. Synchronous video consultation was the most well-known telemedicine activity (86.9%), while asynchronous tele-expertise was the least recognized, according to a study conducted in France to assess knowledge, attitudes, and practices of telemedicine education and training among French medical students and residents (40.3%) [19].

During the pandemic, eLearning was employed as a remote learning strategy in most parts of the world. According to a cross-sectional study of medical students from more than 13 Libyan medical institutions, 75.6% of those surveyed had heard of eLearning and 71.6% had heard of the services given through it. While 813 (24.3%) of the 3,348 individuals had a better comprehension of e-learning than the others, 2,535 (75.7%) had a poor understanding [12].One of descriptive cross-sectional study on students’ perception of E-learning during Covid-19 at a private medical college in Pakistan revealed that 77.4% of students had a negative perception of eLearning [20]. The study shows that; students are still more inclined towards face to face teaching rather than e-teaching. However, in a descriptive web-based cross-sectional study of Nepalese nursing students, it was shown that 58.9% had a favorable attitude toward e-learning. According to the study, the majority of students have a positive view about eLearning when it comes to the usage of distant education [21].In a cross-sectional survey done at Juntendo University Faculty of Medicine in Tokyo, Japan to assess medical students’ understanding of e-learning and attitudes toward it, a substantial majority of medical students (54.2%) favored asynchronous sessions [22]. These finding imply that medical students could have had difficulties transitioning to e-learning.

During the pandemic, eLearning has been employed as a viable medium for distance education delivery at medical institutions, according to a research performed in India on the impact and perception of distant online medical education (tele-education). Undergraduate medical students showed a generally favorable attitude toward eLearning approaches [23].

Continuing medical education, postgraduate education, undergraduate education, and education through participation in telemedicine services are some of the primary categories of tele-education in South Africa, which has progressed in tandem with technology improvements [1].

According to a cross sectional survey conducted in Addis Ababa University, Ethiopia, to assess medical students’ perception on the impact of the “flooding” policy on medical education and e-learning initiatives, it was revealed that most preclinical students (85.1%) who had attended live-streamed lectures preferred traditional classroom lectures, which is due to equipment malfunctions. The study explored that the implementation of e-learning activities has a potential challenge initially. Despite some initial challenges in, it is necessary to continue strengthening medical education in Ethiopia and Sub-Saharan Africa and further exploration of technology to improve education [24].

In Africa, tele-education has been used in different forms in the health sector for the past years. Institutions in Africa have different experiences and perceptions of e-learning initiatives. Evidences show that, in the future, internet-based e-learning in Ethiopia might be more successful [24]. However, it’s crucial to guarantee the quality and functionality of the current e-learning initiative before a new one is fully implemented.

In order to deploy successful e-learning systems in a developing nation, understanding of user characteristics is required in the creation and usage of eLearning systems [25–27]. Thus, the aim of this study will enable us to understand the user’s levels of knowledge and attitude towards tele-education.

## Method and materials

### Study design

An institution-based quantitative cross-sectional study design supported with a qualitative study was conducted to assess Medical Students’ knowledge and attitude towards tele-education at University of Gondar.

### Study area and study period

The study was conducted at the University of Gondar (UOG) from May 5 to June 10, 2022. It is located in Gondar, Ethiopia’s former capital, and was founded in 1954 as the Public Health College. As of 2016, the institution offers 56 undergraduate and 64 graduate programs.

College of Medicine and Health Science(CMHS) is one of the recognized colleges in Ethiopia. The CMHS currently has over 7,000 students, and employs over 1,000 full-time faculty members. The College currently offers 22 undergraduates, 38 postgraduate programs, seven PhD programs, Seven specialty certificate and 3 subspecialties certificate program as of 2022 [34]. From CMHS the school of medicine is one of the pioneers’ schools which enrolled around 1173 MD students [28].

### Populations

All medical students enrolled in the University of Gondar in 2022 was considered as a source of population. The study populations were all medical students from 2nd year to 5th year who were available during data collection at University of Gondar, school of medicine. Students with sick leave during data collection were excluded from the study.

### Sample size determination

The sample size of this study was determined using single population proportion formula:

N = Z_α/2_^2^ *p*(1-p) / d^2^

With the following assumptions:

n = the required sample size.

Z = the value of standard normal distribution corresponding to α/2 = 1.96,

p = proportion of knowledge and attitude of medical students.

q = 1-p = proportion of No knowledge and attitude of medical students.

d = is the margin of error 5% (0.05).

With 95% confidence interval (CI) and a proportion of tele-education knowledge and attitude of 50%, since there is no previous study done in the same population. After taking into account 10% non-response rate. Accordingly, the sample size was 424. For the qualitative part; seven study participants were participated and level of saturation was used to determine the minimum sample size.

### Sampling technique and procedures

Proportionate stratified sampling technique was performed to select medical students from 2nd year to 5th year enrolled 2022. Human Resource list of medical students in each study year was used as a sampling frame to identify potential study participants. We assumed that medical students who are attending the study are heterogeneous with their study year regarding knowledge and attitude towards tele-education.

Study participants were selected randomly using record identification numbers retrieved from the sampling frame from each group based on their study year. Lottery method was used to randomly select a set of medical students as respondents of this study.

For the qualitative study, heterogeneous sampling method was used to select the study participants.

### Variables of the study

The outcome variables were measured dichotomously as attitude towards tele-education (Favorable and Unfavorable) and Knowledge towards tele-education (Good and Poor). Socio demographic characteristics: age, sex (male, female), previous-residence (urban, rural), year of study (2nd -5th ) and self-rated health status, ICT exposures: training related to ICT, computer ownership, knowledge of medical education digitization, Source of information, perception of e-learning technologies and internet access type, Perceived organizational factors: perceived access to computers, perceived access to the internet and computer-related course and Level of literacy: eHealth literacy(eHL) and computer literacy(CL) were the independent variables.

### Operational definition

#### Knowledge of tele-education

respondent’s knowledge about tele-education technologies and use for medical education was assessed. Ten items with “yes” or “no” responses were used to assess knowledge of tele-education. The average score was used as a cutoff point to identify students with good and poor knowledge. Study participants who have scored greater than or equal to 70% of the ten 10 questions was assigned to good knowledge otherwise poor knowledge. The average score value was determined by median average score of students [29].

#### Attitude towards tele-education

respondent’s experience, opinion and overview towards tele-educations relative advantage, compatibility, complexity and trial ability were assessed. A five point Likert scale was used to score the responses ranging from strongly agree 5 to strongly disagree 1. After checking symmetric normality assumptions, since, the sample data is not normally distributed the respondents score was based on the median score. Then by taking the proportion of respondents to answer a set of questions, those students who have scored greater than the median score of each questions (five point Likert scale) of tele-education have favorable attitude and less than the median score of each questions (five point Likert scale) of tele-education have unfavorable attitude [29].

### Data collection tools and procedures

For the quantitative finding the structured questionnaires were used. The questionnaires were adopted and modified from different reviewed literatures. The tool consists of questions about socio-demographic characteristics, perceived organizational factors, ICT exposures and level of literacy. The respondents written informed consent was obtained, the information about how to fill the tool was given by the data collector and a pretested self-administered structured questionnaire was delivered for the medical students to answer. To do this, two supervisor and two data collectors had been recruited and involved for the data collection process.

For the qualitative study the data was collected using in-depth interviews. The questions were prepared to be open ended and not sensitive for political issues. The questionnaires were first developed in English and then underwent forward and backward translation to ensure semantic consistency (English to Amharic then English), for appropriateness and easiness in approaching the study participants. The in-depth interview was conducted with seven medical students selected from each study year randomly. The interview had been held with principal investigator and the student at the University of Gondar (UOG) from May 5 to June 10, 2022.

### Data quality control

One-day training was given for data collectors and supervisors on research ethics, providing informed consent, data collection procedures, data collecting tools, how to approach participants, data confidentiality, respondent’s right and all the study protocols to be followed throughout the course of the data collection period.

Continuous monitoring by supervisors was done throughout data collection period to ensure that the data is collected according to the study protocol. Before the actual data collection, 5% of the sample pretesting of the questionnaire was conducted among medical students of Bahirdar University. The reliability of the data was assessed using Cronbach’s alpha and it was very good for all scale variables with a Cronbach’s alpha value > 0.8.

### Data Processing and Analysis

Data entry template was created based on study variables on Epi data version 4.6. Manually edited data was entered to the software for further editing and exported to Statistical Package for Social Science (SPSS) version 25 for further analysis.

Data was checked, cleaned, edited, and analyzed by using SPSS version 25. Internal consistency was checked using Cronbach’s alpha. Descriptive analysis had been computed. Means and percentages were calculated to describe the profile of the respondents. Binary logistic regression was conducted with one explanatory variable and each dependent variable and a p-value < 0.2 was taken to multivariable analysis. In multivariable analysis the variables with p-value < 0.05 was reported as significant and explanatory variable.

The adjusted odds ratio(AOR) was used to measure the association between the dependent and independent variables. A p-value < 0.05 with 95% confidence interval (CI) was considered statistically significant and presented. Overall model fitness had been checked with the Hosmer–Lemeshow test and the model was fit with p-value > 0.05. The qualitative data which was collected using in-depth interview was transcribed into text in the Amharic language and the Amharic text translated into English, then open code software was used for analysis using content thematic analysis.

## Results

### Socio-demographic characteristic of study participants

A total of 397 medical students were participated in this study with a response rate of 93.63%. More than half 216 (54.4%) of the study participants were male. The mean age of the respondents was 23.07 years (SD ± 1.67) and majority 321 (80.9%) of the study participants were greater than 21 years of age. About 126 (31.7%) of them were fifth year medical students and only 68 (17.1) were from second year. The majority 279 (70.3%) of students were from urban residence. About 333(83.9%) of students rated their health status good (Table 1).

**Table 1:** Socio demographic characteristics of medical students enrolled at University of Gondar, Ethiopia, 2022

Variable	Frequency(%)
Age in year <=21 > 21	76(19.1) 321(80.9)
Sex Male Female	216(54.4) 181(45.6)
Year of study 2nd year 3rd year 4th year 5th year	68(17.1) 88(22.2) 115(29.0) 126(31.7)
Previous residence Urban Rural	279(70.3) 118(29.7)
Self-rated health status Good Fair Bad	333(83.9) 61(15.4) 3(0.8)

### ICT exposure of medical students

The study shows about 296 (74.6%) of students have got training related to computer and information technology. Three hundred seventeen of medical students (79.8%) were have a computer. Of those 237 (74.8%) of them use their computer for internet access and about 130 (54.9%) of them search for information online often. Majority 282 (71. %) of students know about medical education digitization (e-learning, tele-education and simulation technologies) and about 385 (97.0%) of them think that e-learning technologies are very important in their medical education (Table 2).

**Table 2:** ICT exposure of medical students enrolled at University of Gondar, Ethiopia, 2022

Variable	Frequency(%)
Have you got any training related to computer and information technology?	
Yes	296(74.6)
No	101(25.4)
Do you have computer?	
Yes	317(79.8)
No	80(20.2)
What task you perform with your computer?	
For Microsoft	194(61.2)
For internet access	237(74.8)
For entertainment	159(50.2)
For other	52(16.4)
How often do you search for information online?	
Always	53(22.4)
Often	130(54.9)
Sometimes	52(21.9)
Rarely	2(0.8)
Do you think that you have computer skill?	
Yes	295(74.3)
No	102(25.7)
How do you rate your computer skill?	
Beginner	74(25.1)
Average	203(68.8)
Professional	18(6.1)
Do you think e-learning technologies are important in your medical education?	
Yes	385(97.0)
No	12(3.0)
How do you rate the importance of e-learning technologies in medical education?	
Very important	177(46.0)
Important	203(52.7)
Not much important	5(1.3)

### Perceived organizational factor

The perceived organizational factors were aimed to assess how did students perceive ICT related infrastructures from the college. About 193 (48.6%) of study participants think that access to computer from the organization is fair and only 34(8.6%) of them perceive adequate. In this study more than half. 214 (53.9%) of participants perceive that access to the internet provided by the college were also fair and about only 56 (14.1%) of them perceive adequate. The majority 307 (77.3%) of medical students participated in this study have taken computer related course. From those participants about half 157 (51.1%) of them believes that the course they have taken was inadequate in relation to tele-medicine knowledge (Table 3).

**Table 3:** Perceived organizational factors of medical students enrolled at University of Gondar, Ethiopia, 2022

Variables	Frequency(%)
Perceived access to computers provided by the college	
Inadequate	170(42.8)
Fair	193(48.6)
Adequate	34(8.6)
Perceived access to internet provided by the college	
Inadequate	127(32.0)
Fair	214(53.9)
Adequate	56(14.1)
Have taken computer related courses	
Yes	307(77.3)
No	90(22.7)
Rating of the course(s) in relation to tele-medicine knowledge	
Inadequate	157(51.1)
Fair	136(44.3)
Adequate	14(4.6)

### Knowledge of medical students towards Tele-education

In this study more than half 230 (57.9%) of medical students had good knowledge towards tele-education (95% CI: 52%, 62%) and about half 106 (46.1%) of them were female. The majority 308 (77.6%) of students have heard about tele-education and about half. 195 (49.1%) of them know its effect on medical education quality. Most 329 (82.9%) of students considered tele-education as a type of e-learning. In this study about half. 203 (51.1%) of study participants’ believes that tele-education in the medical field is not considered expensive than conventional learning (Table 4).

**Table 4:** Descriptive statistics of medical student’s knowledge towards tele-education at University of Gondar, Ethiopia, 2022

No	Statements	Yes N(%)	No N(%)
1	Have you ever heard about tele-education?	308(77.6)	89(22.4)
2	Have you ever seen tele-education system?	154(38.8)	243(61.2)
3	I know tele-education technology	158(39.8)	239(60.2)
4	I know the effect of tele-education on medical education quality	195(49.1)	202(50.9)
5	Tele-education depends on a comprehensive digital electronic environment displaying educational curriculum through electronic networks	309(77.8)	88(22.2)
6	Tele-education is an interactive system that provides an opportunity for learning through Information and Telecommunication Technology	325(81.9)	72(18.1)
7	Tele-education in the medical field is not considered less expensive than conventional learning	194(48.9)	203(51.1)
8	Tele-education provides a digital multimedia content (written text, audio, video and images)	317(79.8)	80(20.2)
9	One of the benefits of Tele-education with live content is that the scholar receives instant feedback from the instructor	284(71.5)	113(28.5)
10	Tele-education is considered a type of e-learning	329(82.9)	68(17.1)

### Factors associated with medical student’s knowledge towards Tele-education

The bivirable logistic regression was assessed among the dependent variable and each independent variable. The variables with p-value < 0.2 were taken to multivariable logistic regression analysis. As shown in (Table 5) the multivariable logistic regression revealed that taking training related to information, communication and technology, knowledge of medical education digitization, computer literacy and attitude towards tele-education have a significant association with the students’ knowledge towards tele-education.

**Table 5:** Factors associated with medical student’s knowledge towards tele-education at University of Gondar, Ethiopia,2022

Variable	Knowledge		95% CI	
	Good N(%)	Poor N(%)	COR	AOR
Age				
<=21 >21	26(6.55) 204(51.38)	50(12.59) 117(29.5)	1.0 0.29(0.17,0.50)*	1.0 0.99(0.35,2.82)
Year of study				
2nd year 3rd year 4th year 5th year	21(5.28) 48(12.09) 66(16.62) 95(23.93)	47(11.84) 40(10.08) 49(12.34) 31(7.808)	1.0 2.68(1.38,5.21) * 3.01(1.60,5.68) * 6.85(3.56,13.20)*	1.0 2.23(0.77,6.42) 2.55(0.78,8.32) 3.17(0.94,10.61)
Training related to ICT				
Yes No	199(50.13) 31(7.8)	97(24.43) 70(17.63)	4.63(2.84,7.54) * 1.0	2.27(1.13,4.55) ** 1.0
Computer ownership				
Yes No	195(49.12) 35(8.82)	122(30.7) 45(11.34)	2.05(1.25,3.37) * 1.0	1.37(0.72,2.60) 1.0
Knowledge of medical education digitization technologies				
Yes No	199(50.13) 31(7.8)	83(20.9) 84(21.16)	6.49(4.00,10.55) * 1.0	3.80(2.12,6.84) ** 1.0
Perceived importance of e-learning				
Yes No	229(57.7) 1(0.25)	156(39.3) 11(2.77)	16.14(2.06,126.33) * 1.0	4.06(0.47,35.25) 1.0
Computer related course(s)				
Yes No	196(49.4) 34(8.6)	111(27.9) 56(14.1)	2.90(1.79,4.72) * 1.0	0.73(0.35,1.52) 1.0
eHL				
High Low	151(38.04) 79(19.9)	64(16.1) 103(25.9)	3.07(2.03,4.65) * 1.0	1.23(0.72,2.11) 1.0
CL				
High Low	160(40.3) 70(17.6)	58(14.61) 109(27.5)	4.29(2.80,6.56) * 1.0	2.82(1.68,4.72) ** 1.0
Attitude of tele-education				
Favorable Unfavorable	159(40.1) 71(17.9)	52(13.1) 115(28.9)	4.95(3.31,7.61) * 1.0	3.52(2.12,5.84) ** 1.0

N.B: statistically significant *p-value < = 0.05 for bivirable analysis, **p-value < 0.05 for multivariable analysis, eHL: electronic health literacy, CL: computer literacy

The study shows that taking ICT related trainings has a significant association with the knowledge of tele-education. Study participants who have got training related to Information Communication and Technology were about 2.27 times more likely to have good knowledge towards tele-education as compared to those who did not take training (AOR = 2.27 95% CI; (1.13,4.55)) (Table 5).

With related to ICT exposure; medical students who know medical education digitization technologies; e-learning, tele-education and simulation technologies were 3.8 times more likely have good knowledge towards tele-education as compared to those who do not know (AOR = 3.80 95% CI; (2.12,6.84)). A student who are high in computer literacy were 2.82 times more likely to have good knowledge about tele-education than those with low computer literacy (AOR = 2.82 95% CI; (1.68,4.72)) (Table 5).

The study also shows that the students who have favorable attitude towards tele-education were 3.52 times more likely have good knowledge to tele-education as compared to those who have unfavorable attitude towards tele-education after controlling for all other factors (AOR = 3.52 95% CI; (2.12,5.84)) (Table 5).

### Attitude of medical students towards tele-education

The study revealed that more than half 211 (53.1%) of medical students participated on the study had a favorable attitude towards tele-education (95% CI: 48%, 57.9%). Out of study participants about 186 (88.2%) of them were greater than 21 years of age and more than half 120 (56.9%) of them were male. From those who had a favorable attitude about 159 (75.4%) of them had good knowledge towards tele-education. Most 315 (79.4%) of study participants believes that many educational problems can be solved using tele education platform and about 308 (77.6%) of them also believes that it can save time. This study shows that about 296 (74.5%) of them believes that tele-education improves access to materials. Almost half 175 (44.1%) of medical students participated in this study suggests that tele-education helps them to achieve better results and about half 199 (50.1%) of them think that to reinforce their knowledge tele-education help them. In this study 299 (75,3%) of them like the idea of using tele education. Hence, about half 205 (51.6%) of them suggests that the universities should adapt tele education (Table 6).

**Table 6:** Descriptive statistics of medical students’ attitude towards tele-education at University of Gondar, Ethiopia, 2022

S.No	Statements	SA N(%)	A N(%)	NE N(%)	D N(%)	SD N(%)
1	Tele education can solve many of educational problem	129(32.5)	186(46.9)	75(18.9)	7(1.8)	0
2	Tele education saves time	113(28.5)	195(49.1)	82(20.7)	6(1.5)	1(0.3)
3	Tele education improves access to materials	112(28.2)	184(46.3)	94(23.7)	5(1.3)	2(0.5)
4	Tele education helps me to achieve better results	85(21.4)	175(44.1)	123(31.0)	13(3.3)	1(0.3)
5	Tele education increases learners engagement	83((20.9)	168(42.3)	120(30.2)	25(6.3)	1(0.3)
6	Tele education improves interaction between teachers and students	57(14.4)	126(31.7)	152(38.3)	52(13.1)	10(2.5)
7	Tele education increases my understanding of concepts	74(18.6)	166(41.8)	136(34.3)	20(5.0)	1(0.3)
8	Tele education has created more problems than it solved	29(7.3)	53(13.4)	187(47.1)	97(24.4)	31(7.8)
9	Tele education is too time consuming to use	60(6.0)	72(18.1)	148(37.3)	114(28.7)	39(9.8)
10	Tele education has had little impact on me	23(5.8)	97(24.4)	169(42.6)	81(20.4)	21(6.8)
11	Tele education as informative as teachers	39(9.8)	116(29.2)	168(42.3)	62(15.6)	12(3.0)
12	Tele education will never replace other forms of teaching and learning	40(10.1)	104(26.2)	162(40.8)	76(19.1)	15(3.8)
13	Tele education help to reinforce my knowledge	80(20.2)	199(50.1)	110(27.7)	7(1.8)	1(0.3)
14	Tele education helps me to catch up missed lectures	78(19.6)	185(46.6)	120(30.2)	12(3.0)	2(0.5)
15	Universities should adapt for their students tele education	105(26.4)	205(51.6)	76(19.1)	8(2.0)	3(0.8)
16	I like the idea of using tele education	104(26.2)	195(49.1)	90(22.7)	7(1.8)	1(0.3)
17	Using tele education makes learning fun.	88(22.2)	188(47.4)	111(28.0)	9(2.3)	1(0.3)
18	My institute has an updated website for tele-education	43(10.8)	117(29.5)	176(44.3)	39(9.8)	22(5.5)
19	My institute has adequate technology for tele education.	41(10.3)	101(25.4)	173(43.6)	55(13.9)	27(6.8)
20	Technical assistance is necessary from college support services for tele-education.	69(17.4)	151(38.0)	119(30.0)	43(10.8)	15(3.8)
21	Slow internet connections stress me.	142(35.8)	155(39.0)	83(20.9)	12(3.0)	5(1.3)
22	Tele education should be used to reduce travel related stress	136(34.3)	165(41.6)	84(21.2)	11(2.8)	1(0.3)

N.B: SA: Strongly agree A: Agree D: Disagree NE: Neutral SD: Strongly disagree.

### Factors associated with medical student’s attitude towards tele-education

The bivirable logistic regression was assessed to determine the association between the dependent variable attitude towards tele-education and each independent variable. As it was shown in (Table 7) in this study age, year of study, training related to ICT, knowledge of medical education digitization technologies, perceived importance of e-learning, taking computer related courses, e-health literacy, computer literacy and knowledge towards tele-education of medical students were associated significantly with attitudes towards tele-education with p-value < 0.2 in bivirable logistic regression.

**Table 7:** Factors associated with medical student’s attitudes towards tele-education at University of Gondar, Ethiopia,2022

Variable	Attitude		95% CI	
	Favorable N(%)	Unfavorable N(%)	COR	AOR
Age				
<=21 >21	25(6.3) 186(46.9)	51(12.9) 135(34.0)	1.0 2.81(1.65,4.76)*	1.0 3.89(1.33,11.39)**
Year of study				
2nd year 3rd year 4th year 5th year	27(6.8) 42(10.6) 61(15.4) 81(20.4)	41(10.3) 46(11.6) 54(13.6) 45(11.3)	1.0 1.38(0.73,2.63) 1.71(0.93,3.15) 2.73(1.48,5.01)*	1.0 0.36(0.12,1.1) 0.34(0.11,1.15) 0.36(0.11,1.25)
Training related to ICT				
Yes No	173(43.6) 38(9.6)	123(30.9) 63(15.9)	2.33(1.46,3.70) * 1.0	1.16(0.62,2.18) 1.0
Knowledge of medical education digitization technologies				
Yes No	171(43.1) 40(10.1)	111(27.9) 75(18.9)	2.88(1.83,4.53) * 1.0	1.45(0.84,2.51) 1.0
Perceived importance of e-learning				
Yes No	210(52.9) 1(0.25)	175(44.1) 11(2.8)	13.20(1.68,103.24) * 1.0	4.16(0.51,34.04) 1.0
Computer related course(s)				
Yes No	177(44.6) 34(8.6)	130(32.8) 56(14.1)	2.24(1.38,3.63) * 1.0	1.19(0.63,2.27) 1.0
eHL				
High Low	137(34.5) 74(18.64)	78(19.6) 108(27.2)	2.56(1.70,3.84) * 1.0	1.45(0.88,2.36) 1.0
CL				
High Low	133(33.5) 78(19.6)	85(21.4) 101(25.4)	2.02(1.35,3.02) * 1.0	0.99(0.61,1.63) 1.0
Knowledge of tele-education				
Good Poor	159(40.1) 52(13.1)	71(17.9) 115(28.9)	4.95(3.21,7.61) * 1.0	3.42(2.06,5.66) ** 1.0

N.B: statistically significant *p-value < = 0.05 for bivirable analysis, **p-value < 0.05 for multivariable analysis, eHL: electronic health literacy, CL: computer literacy

The multivariable logistic regression reveals that age group and knowledge towards tele-education of study participants were associated significantly with attitude towards tele-education (Table 7).

The study shows that medical students with greater than 21 age group were 3.89 times more likely to have a favorable attitude towards tele-education as compared to those students with less than 21 years of age group (AOR = 3.89, 95% CI; (1.33,11.39)) (Table 7).

In this study the medical students who have a good knowledge towards tele-education were 3.42 times more likely to have a favorable attitude than those students who have a poor knowledge towards tele-education (AOR = 3.42, 95% CI; (2.06,5.66)) (Table 7).

### Qualitative findings

Seven medical students from University of Gondar participated in In-depth interview until information saturation was reached, in order to explore their attitude towards tele-education. From second to the fifth study year, students were chosen. The study used a phenomenological approach which helps to assess how study participants actually experienced tele-education. Study participants’ information was organized into three main themes. These themes include the importance of tele-education, tele-education prerequisites and tele-education challenges.

The medical students who were interviewed were agreed that tele-education provides a valuable support for medical education. Tele-education provides an option for medical students to continue education and to maintain their qualifications and certifications. Video oriented lessons from an experts were indicated to be more important for having standardized and brief understanding in their course matter. One of a 24 years old 4th year medical student explains the benefits of tele-education in the following way *“it probably helps us to get a standardized lesson because it can show us the process and everything”.* Tele-education services were indicated to be mandatory in which distance is major factor. A 25 years old 5th year medical student explores this in such a way “*I think it is not only important, it is obligatory. It is well important”.*

Study participants revealed that tele-education can update the existing educational system to better. A 23 years old student from 4th year explains the purpose of tele-education in the following way “*its main purpose is to update the existing educational system, to increase the transfer of knowledge from the teacher to the student. In this way, it is to enrich the student and produce a competent, knowledgeable and skilled student. ….’*. According to study participants’ explanation there is a mismatch between medical students’ learning needs and the realities of today’s clinical settings. Study participants’ revealed that it may be difficult in the country’s setup and economy to have standard medical education. Which is mainly due to a lack of available medical education equipment and expertise. ”…. *when they show us practical lessons in foreign countries where there is no shortage of equipment and expertise, we develop practical knowledge.”*

The study participants’ also emphasized the need for tele-education requirements. The participants in the study looked at a variety of difficulties that needed to be resolved at the individual, institutional, and governmental levels for the effective use of tele-education for medical education purposes. One of study participant from 4th year says” *the first thing is to narrow the gap in the middle of; what is called availability. First, in terms of getting the materials for education. Second, in terms of training the users. In order to narrow it down, bringing the materials that are useful for tele-education and training people on how to use it.*”

The study participants also discuss the negative aspects of using tele-education services. They are concerned that employing e-learning tools instead of face-to-face instruction could result in the loss of crucial learning features. One of the study participant from 5th year medical class explains his concerns in using tele-education services for medical education in such a way “*…As a medical science education, we need to practice physically what we have learned to understand it in detail. We have to see, touch and evaluate a patient in person. It will give us a different mental exposure. But, in video oriented lessons we lack this. “*.

In general, the study participants’ in this study believes that tele-education services are crucial to the quality of medical education; because they allow them to access expensive and significant study materials from abroad, share knowledge with international classes, and gain advanced skills and knowledge. They consider tele-education to be a crucial and important component of distance learning. Moreover, they have recommended that the government have to design a policy for e-learning use in medical education, the institution should have to use infrastructure’s effectively for use of e-learning and there should be a training and awareness creation for students.

## Discussion

The use of multimedia tools in particular have improved online and distance learning methods. It is realistic to assume that tele-medical education will quickly become a crucial component of the instruction for the majority of health professionals, enhancing their access to a variety of top-notch courses [30].

The finding of this study reveals that 57.9% of study participants had good knowledge about tele-education (95% CI: 52%, 62%). In this study as a knowledge output the majority 77.6% of students have heard about tele-education, a study conducted in Uganda among undergraduate medicine and nursing students at Makerere University, which also reveals that a vast majority 96% of the students had heard about e-learning [17]. This might be that the idea of e-learning initiatives in the higher education is in action.

Furthermore, the finding of this study revealed that 53.1% of medical students participated on the study had a favorable attitude towards tele-education (95% CI: 48%, 57.9%). The finding is in line with study conducted in Libya of Nepalese nursing students and Sudan [21, 31].This result is also supported with the qualitative finding. A 22 years old student from 3rd year said “*I believe that tele education will improve the quality of education to a better level, because we use different technologies… they will help us to develop skills by looking at different graphics in a short period of time. I think that it is a platform where you can buy the so-called better knowledge in a short time.”*

Taking ICT related trainings has a significant association with the knowledge of tele-education. Study participants who have got training related to Information Communication and Technology were about 2.27 times more likely to have good knowledge towards tele-education as compared to those who do not taken training (AOR = 2.27 95% CI; (1.13,4.55)). It is supported with study conducted in Iran at Mashhad University of Medical Sciences (MUMS) which suggests that, it was advised to put training programs in place, such as workshops, to help students become aware of and proficient with using e-learning as a tool for learning [32]. Findings show that education and training typically alter people’s perspectives, perceptions and understandings [33].

In this study medical students who know medical education digitization technologies were 3.8 times more likely to have good knowledge towards tele-education as compared to those who do not know (AOR = 3.80 95% CI; (2.12,6.84)). This might be the result of curiosity about recent technical developments [30]. This finding is also supported with the qualitative result. One of the participants from 4th year said that “*Being ready can be with knowledge, it can be with money, we just need to be able to prepare ourselves with different things. And we must be able to know the things related to this technology thoroughly, from the beginning to the end, we must be able to know the details. Knowing this will be useful for many things*”.

The study indicates that students who are high in computer literacy(CL) were 2.82 times more likely to have good knowledge about tele-education than those with low computer literacy (AOR = 2.82 95% CI; (1.68,4.72)). As it was revealed; due to the fast evolving information culture, CL is an urgent demand for medical and paramedical students. Finding shows that, for information technology applications like telemedicine and tele-education in health care and medical education, computer literacy is a good indicator of success [34].

Medical students who have a good knowledge towards tele-education were 3.42 times more likely to have a favorable attitude than those students who have a poor knowledge towards tele-education (AOR = 3.42, 95% CI; (2.06,5.66)). This could be because, having understanding of the advantages and disadvantages of technological services is essential for having a positive perspective, as indicated in findings [30]. This finding is also supported with qualitative result. One of a 2nd year medical student said that “*The student needs to have a well-computerized knowledge in order to accept it first. So, to acquire knowledge about existing technologies, to have a computerized knowledge, to encourage him to use his knowledge, and to promote the good things that have been acquired through the use of tele technology is good*”.

In this study it was revealed that other socio demographic factors like sex, residence and study year have no association with attitude towards tele-education. The study shows that the difference in the age group between study participants towards tele-education attitude, which is similar to other finding done before [35].

In this study it was revealed that there is no difference in between male and female student’s attitude towards tele-education similar with study conducted in Libya of Nepalese nursing students [21]. However, different from the study conducted in Pakistani which suggests that male students in Pakistani higher education have a more favorable attitude toward e-learning than female students and a study conducted in students’ knowledge and attitude towards E- learning in Mashhad University of Medical Sciences (MUMS) of Iran; which shows a difference in sex [32, 36].

### Strength and limitations of the study

This study is unique in Ethiopia because it discovered medical students’ knowledge and attitude towards tele-education. In addition to this the study was supported by qualitative finding. However, the study had some limitations. Self-reported data were used in this study, which might have introduced self-report bias into survey results. The study’s other drawback is that it was only carried out in one institution. The results could not be generalized to other Ethiopian medical schools. Since, different e-learning initiatives are used and implemented differently.

## Conclusion

The study generally revealed that the knowledge of the medical students about tele-education at the University of Gondar was good. Nearly five out of ten of them also had a favorable attitude towards tele-education. In this study it was shown that, taking training related to information, communication and technology, knowing of medical education digitization, computer literacy and attitude towards tele-education have a significant association with students’ knowledge towards tele-education. Additionally, the study reveals that age group and knowledge towards tele-education of study participants were associated significantly with attitude towards tele-education.

### Recommendation

The implementation of e-learning initiatives for medical education should be enhanced. To preserve the current educational system secure and undisturbed, improvements should be made as smoothly as possible. The medical school at this university must effectively utilize its resources to provide instruction using cutting-edge technological materials. To share knowledge and advanced resources and to provide students with a sufficient education, there should be a cooperative link or relationship with other established medical schools abroad. The institution must also educate the students on the advantages of e-learning programs for medical education. Therefore, in order to employ e-learning initiatives effectively for medical education purposes, higher education institutions must create awareness of them and provide training on their usage and benefits.

## Data Availability

All relevant data are in the manuscript. However, the minimal data underlying all the findings in the manuscript will be available upon request to the corresponding author (rorisaroar@gmail.com).

## Abbreviations

AOR: Adjusted Odds Ratio
COVID 19: Corona Viral Disease 2019
COR: Crude Odds Ratio
CI: Confidence Interval
EMSA: Ethiopian Medical Student’s Association
ICT: Information Communication Technology
IRB: Institutional Review Board
SPSS: Statistical Package for Social Science
TM: Telemedicine
UOG: University of Gondar
